# A New Ilarvirus Found in French Hydrangea

**DOI:** 10.3390/plants11070944

**Published:** 2022-03-30

**Authors:** Giuseppe Parrella, Elisa Troiano

**Affiliations:** Institute for Sustainable Plant Protection of the National Research Council (IPSP-CNR), Piazzale Enrico Fermi 1, 80055 Portici, NA, Italy; elisa.troiano@ipsp.cnr.it

**Keywords:** Ilarvirus, plant RNA virus, virus detection, *Hydrangea macrophylla*, subgroup 2

## Abstract

In this study, a new virus was identified in French hydrangea plants, exhibiting chlorotic vein banding and necrotic ring spots on older leaves. The virus was mechanically transmitted to herbaceous hosts, in which it induced local and systemic or only local symptoms. The genome of the new virus was characterized and consisted of three RNA sequences that were 3422 (RNA 1), 2905 (RNA 2) and 2299 (RNA 3) nucleotides long, with five predicted open reading frames; RNA2 was bicistronic and contained conserved domains and motifs typical of ilarviruses. The phylogenetic analysis of the predicted proteins—p1, p2a, p3a and p3b—revealed its close relationship to recognized members of subgroup 2 within the genus *Ilarvirus*. Homologous antiserum was effective in the detection of the virus in plant extracts and no cross reactions with two other distinct members of subgroup 2 were observed. Overall, the biological features, phylogenetic relationships and serological data suggest that this virus is a new member of the genus, for which we propose the name hydrangea vein banding virus (HdVBV).

## 1. Introduction

The genus *Hydrangea* L. (family *Hydrangeaceae* Dumort) encompasses 80 species that are native to a wide range of regions and countries, including Japan, Asia, Indonesia, the Himalayan mountains and the Americas [1]. Of these, *Hydrangea macrophylla*, also known as French hydrangea or bigleaf hydrangea and here referred to simply as hydrangea, is the most popular cultivated species, with over 1000 recognized cultivars and varieties with various shapes, sizes and bloom colors that are grown in both temperate and sub-tropical climates [2].

Viral diseases may have a significant impact on the appearance, health and market value of hydrangea, especially because it is propagated vegetatively and viruses are easily transmitted and accumulated over time through cuttings, causing a decline in the crop. Moreover, in recent years, emerging viral diseases in hydrangea with the capacity to cause more serious yield losses have been described; thus, in addition to phytoplasma diseases, they are considered a serious developing threat to hydrangea [3]. In total, fourteen viruses have been found to infect hydrangeas [4].

**Figure 1:**
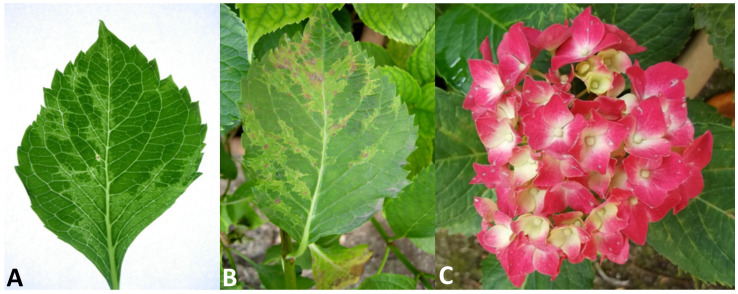
Symptoms associated with HdVBV in *H. macrophylla.* (**A**) Vein banding on an asymmetric young leaf. (**B**) Vein banding and necrotic spots on an older leaf. (**C**) White spots/lines on the floral bracts.

Since the spring of 2016, severe symptoms of leaf deformation, chlorotic vein banding and a mosaic of light and dark green colors (Figure 1A) have been observed in hydrangea plants growing in a private garden of the municipality of Napoli in the Campania region of southern Italy. The chlorotic leaf areas became more evident in older leaves, where necrotic spots and lines also appeared (Figure 1B). On the floral red bracts, some small white spots and short lines were also noticed (Figure 1C).

Here, we will demonstrate that the disease observed in the hydrangea was caused by a novel *Ilarvirus*, which was provisionally designated as ‘hydrangea vein banding virus‘ (HdVBV), based on the main symptom observed on the leaves. Due to the molecular features of HdVBV and its phylogenetic relationships with the other members of the genus, it was assigned to subgroup 2, along with asparagus virus 2 (AV2), citrus leaf rugose virus (CLRV), citrus variegation virus (CVV), elm mottle virus (EMV), lilac ring mottle virus (LRMV), spinach latent virus (SpLV), Tulare apple mosaic virus (TAMV) and tomato necrotic spot virus (ToNSV) [5].

## 2. Results

### 2.1. Symptomatology

**Figure 2:**
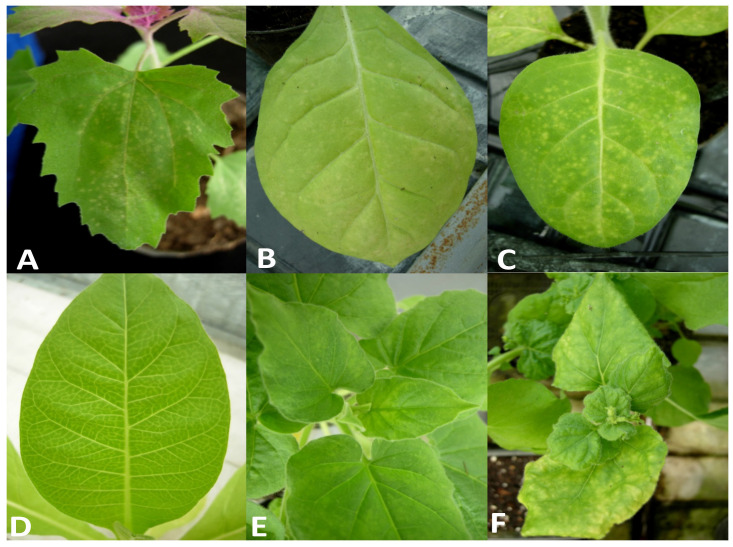
Symptoms induced by HdVBV in some herbaceous hosts: (**A**) pinpoint chlorotic local lesion in *C. amaranticolor*; (**B**) some chlorotic local lesions in *N. tabacum*; (**C**) chlorotic local lesions in *N. rustica*; (**D**) vein clearing in *N. tabacum*; (**E**) mosaic in *N. glutinosa*; (**F**) yellowing and distortion of the apical leaves in *N. benthamiana*.

The symptoms observed on infected hydrangea plants varied slightly depending on the time of observation. In the springtime, we observed light chlorotic vein banding on the youngest leaves (Figure 1A) that became more evident later in the season, at which point ringspots that remained confined within the chlorotic areas also developed (Figure 1B). In addition, some white spots around 1–2 mm in diameter were observed on the red sepals of this variety. The affected leaves were mostly deformed, smaller and had altered symmetry. During the summer, the symptoms appeared to be attenuated and the new leaves were symptomless. In the host range bioassays, five out of the nine tested plant species/varieties were susceptible to the new ilarvirus discovered in hydrangea, as evidenced by the results obtained via degenerate PCR to detect ten distinct virus taxa (see Section 4.4). On the inoculated leaves of *Chenopodium amaranticolor*, symptoms included pinpoint chlorotic local lesions (Figure 2A) that appeared 5 days post-inoculation (dpi) without systemic symptoms. On the inoculated leaves of all *Nicotiana* spp., chlorotic local lesions of 1–2 mm in diameter appeared at 7 dpi (Figure 2B,C), followed by different systemic symptoms, including vein clearing in *N. tabacum* (Figure 2D); light mosaic in *N. glutinosa* (Figure 2E); mosaic, yellowing and leaf area reduction and distortions in *N. benthamiana* (Figure 2F); and light yellowing in *N. rustica* (not shown). No further symptoms were recorded in the other inoculated plants within 60 dpi, and no symptoms were induced in back-inoculated *C. amaranticolor* plants.

Of the three plants grafted with buds taken from a symptomatic plant, only one reproduced similar foliar symptoms to those observed on the symptomatic donor plant (not shown). The symptomatic leaves of this plant produced positive results in RT-PCR with degenerate primers for ilarviruses, and the subsequent sequencing of the amplicon showed 100% nucleotide identity with the sequence of the new ilarvirus, indicating that Koch’s postulates were met.

### 2.2. Nucleotide Sequence and Genome Organization

**Figure 3:**
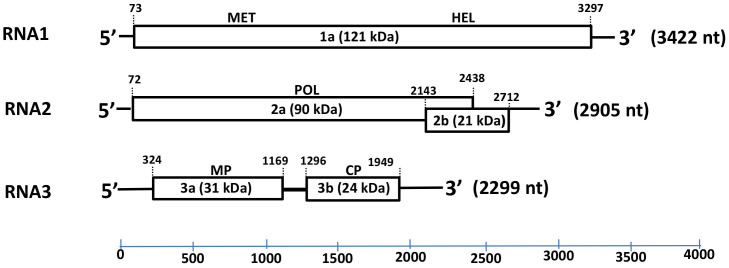
Schematic representation of the hydrangea vein banding virus genome. The expression product of each RNA sequence is presented as rectangular box, whereas lines depict untranslated genomic regions (UTRs). The length of each RNA segment along with the estimated molecular mass (kDa) and function of each putative protein (1a, 2a, 3a and 3b) are indicated. Abbreviations: MET—methyltransferase; HEL—helicase; POL—RNA-dependent RNA polymerase (RdRp); MP—movement protein; CP—coat protein.

The complete genomic sequence of HbVBV, obtained from genome walking and RACE-PCR, consisted of three ssRNA segments of 3422 nucleotides (nt) (RNA 1), 2905 nt (RNA 2) and 2299 nt (RNA 3) in length (Figure 3); the sequences were deposited in the GenBank database (accession nos. OK666835-OK666837) with the provisional name “hydrangea vein banding virus” (HdVBV). The first 44 nt of the RNA 1 and RNA 2–5′ untranslated regions (UTRs) are identical, as well the last 32 nt of the 3′ UTR of all three RNA segments. A sequence analysis suggested that HbVBV has five open reading frames (ORFs; Figure 3).

RNA1 contains a single ORF with the ATG codon at position 73 and ending with a TAG codon at position 3297; it encodes a 121 kDa protein (p1) consisting of 1074 amino acids (AAs). The predicted protein has two conserved domains: a methyltransferase domain between residues 59 and 428 and a helicase domain between residues 769 and 1020. The protein shares 71–91% AA identity and 80–95% similarity with ilarviruses belonging to subgroup 2 (Table 1).

RNA2 is bicistronic with the two ORFs overlapping by 274 nt. The first ORF at the 5′ end of RNA2 encodes for a 788 AA protein (p2a) with a predicted molecular mass of 90 kD, whereas the second ORF encodes a 189 AA protein (p2b) with a predicted molecular mass of 21 kDa. The p2a protein has signature RNA-dependent RNA polymerase (RdRp) motifs between residues 225 and 666 [6], and shares 60–90% AA identity and 72–93% similarity with ilarviruses belonging to subgroup II, while the p2b protein shares 54–85% identity and 70–90% similarity with its orthologs in subgroup II (Table 1).

**Figure 4:**
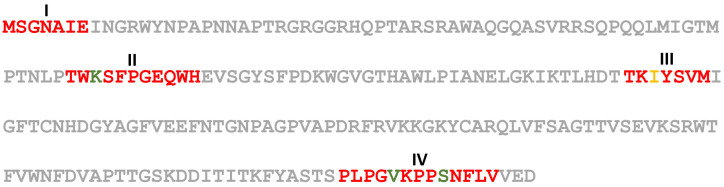
The amino acid sequence of the hydrangea vein banding virus (HdVBV) coat protein. I–IV, conserved amino acid motifs within subgroup 2: red fonts indicate the AAs shared by all ilarviruses within subgroup 2; green fonts identify the variable residues; orange font shows an unexpected residue in the HdVBV coat protein. In addition, motif III has a missing tryptophan residue (W), since the aa sequence shared by all members of subgroup 2 is TKVYSWVM [10].

RNA3 contains two ORFs: the ORF 3a encodes a 281 AA protein (p3a) with an estimated molecular mass of 31 kD and has a signature motif between residues 5 and 244 that identifies the *Bromovirus* movement protein family [7]. The p3a protein has 30–91% identity and 48–95% similarity with the movement proteins of other ilarvirus members belonging to subgroup II (Table 1). The ORF 3b encodes a 217 AA long protein (p3b) with a predicted molecular mass of 24 kD; p3b has a signature motif between residues 21 and 217 that is recognized as an ilarvirus coat protein [8,9]. The p3b protein shares 37–81% identity and 49–88% similarity with the coat protein of other ilarvirus members belonging to subgroup II (Table 1) and shares the four AA motifs present in all members of subgroup II; however, motif III has a single AA substitution (I, isoleucine to V, valine) and one AA deletion (W, tryptophan) (Figure 4) [10].

### 2.3. Phylogenetic Relationships

**Figure 5:**
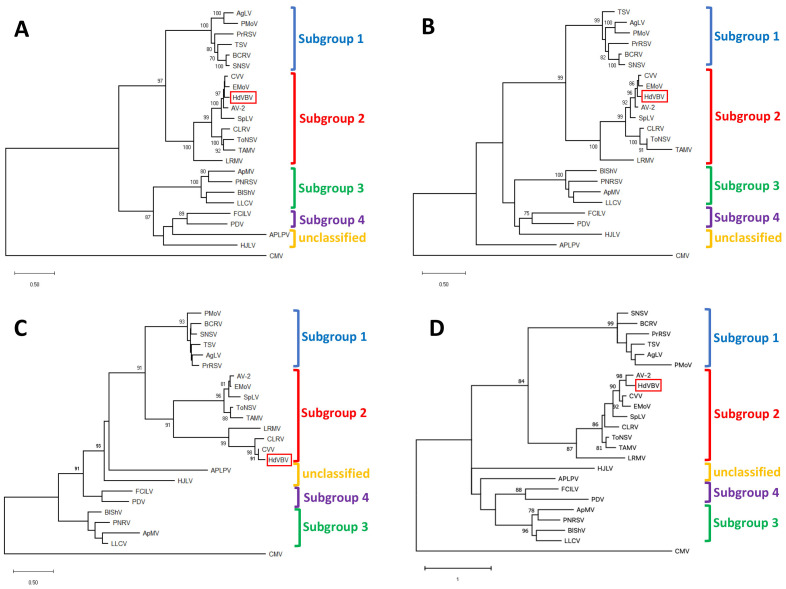
Phylogenetic relationships of hydrangea vein banding virus (HdVBV; GenBank accession nos. OK666835-OK666837) and officially recognized species in the genus *Ilarvirus* based on the alignment of the complete amino acid sequences of p1 (**A**), p2 (**B**), p3a (**C**) and p3b (**D**). Phylogenetic analysis was performed using the maximum likelihood method in MEGA X with 1000 bootstrap replicates based on LG+G (for p1, p2 and p3a) and WAG+G (for p3b) substitution models [11,12]. Bootstrap values >70% at each node are shown. The position of HdVBV within the trees is highlighted with a red box. cucumber mosaic virus (CMV, genus *Cucumovirus*; RNA1: NC_002034; RNA2: NC_002035; RNA3: NC_001440) was used as an outgroup. Viruses and their relative reference sequences used for tree reconstruction are: ageratum latent virus (*AgLV*, RNA1: NC_022127; RNA2: NC_022128; RNA3: NC_022129), American plum line pattern virus (*APLPV*, RNA1: NC_003451; RNA2: NC_003452; RNA3: NC_003453), apple mosaic virus (*ApMV*, RNA1: NC_003464; RNA2: NC_003465; RNA3: NC_003480), asparagus virus 2 (*AV-2*, RNA1: NC_011808; RNA2: NC_011809; RNA3: NC_011807), blackberry chlorotic ringspot virus (*BCRV*, RNA1: NC_011553; RNA2: NC_011554; RNA3: NC_011555), blueberry shock virus (*BlShV*, RNA1: NC_022250; RNA2: NC_022251; RNA3: NC_022252), citrus leaf rugose virus (*CLRV*, RNA1: NC_003548; RNA2: NC_003547; RNA3: NC_003546), citrus variegation virus (*CVV*, RNA1: NC_009537; RNA2: NC_009538; RNA2: NC_009536), elm mottle virus (*EMoV*, RNA1: NC_003569; RNA2: NC_003568; RNA3: NC003570), fragaria chiloensis latent virus (*FCILV*, RNA1: NC_006566; RNA2: NC_006567; RNA3: NC_006568), humulus japonicus latent virus (*HJLV*, RNA1: NC_006064; RNA2: NC_006065; RNA3: NC_006066), lilac leaf chlorosis virus (*LLCV*, RNA1: NC_025477; RNA2: NC_025478; RNA3: NC_025481), lilac ring mottle virus (*LRMV*, RNA1: EU919668, partial sequence; RNA2: NC_038777; RNA3: 038776), parietaria mottle virus (*PMoV*, RNA1: NC_005848; RNA2: NC_005849; RNA3: NC_005854), privet ring spot virus (*PrRSV*, RNA1:NC_027928; RNA2: NC_027929; RNA3: NC_027930), prune dwarf virus (*PDV*, RNA1: NC_008039; RNA2: NC_008037; RNA3: NC_008038), prunus necrotic ringspot virus (*PNRV*, RNA1: NC_004362; RNA2: NC_004363; RNA3: NC_004364), spinach latent virus (*SpLV*, RNA1: NC_003808; RNA2: NC_003809; NC: 003810), strawberry necrotic shock virus (*SNSV*, RNA1: NC_008708; RNA2: NC_008707; RNA3: NC_008706), tobacco streak virus (*TSV*, RNA1: NC_003844; RNA2: NC_003842; RNA3: NC_003845), tomato necrotic streak virus (*ToNSV*, RNA1: NC_039074; RNA2: NC_039075; RNA3: NC_039076) and Tulare apple mosaic virus (*TAMV*, RNA1: NC_003833; RNA2: NC_003834; RNA3: NC_003835).

Maximum likelihood-based phylogenetic analyses of all the ORFs, performed with MEGA X under the best fit substitution models, always placed HdVBV in a well-supported clade comprising the recognized members of subgroup II in the genus *Ilarvirus*. These results were confirmed by a high bootstrap value in all instances (Figure 5).

### 2.4. Electron Microscopy, Coat Protein Mass Determination and Serological Detection

**Figure 6:**
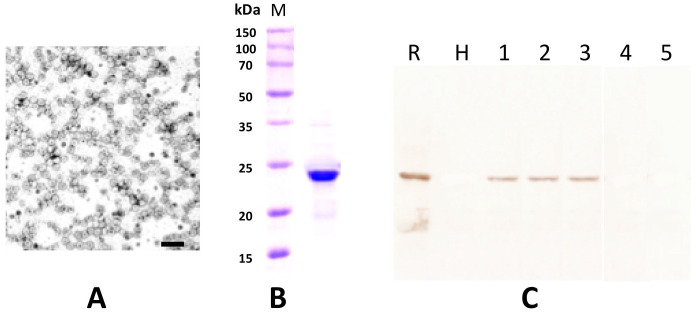
(**A**) Partial purified virus preparation obtained from symptomatic leaves of hydrangea (the bar corresponds to 100 nm). (**B**) Estimation of the coat protein molecular mass using the partial purified virus preparation in 12.5% SDS-PAGE. Lane M shows the marker protein profile. The molecular weights of the marker proteins are reported on the left side of the gel. (**C**) Western blot detection of HdVBV coat protein in healthy and diseased plants with antibodies elicited by the recombinant His-coat protein. Lane R shows the recombinant His-coat protein used to elicit antibodies; lane H shows extract from a symptomless hydrangea plant; lanes 1–3 show extracts from diseased hydrangea plants; lane 4 shows extract from a plant infected with spinach latent virus (SpLV); lane 5 shows extract from a plant infected with asparagus virus 2 (AV-2).

The partial purified virus preparations contained quasi-isometric particles with a mean diameter of 30 nm and some heterogeneity in size, as is typically reported for ilarviruses (Figure 6A) [5]. From the His-tagged CP soluble fraction that was recovered from *E. coli* transformed with pET-30a-CP, only a protein band with a molecular mass of 24 kD was clearly visible in 12.5% SDS-PAGE after purification using a Ni-NTA agarose column and dialysis in 145 mM NaCl solution (Figure 6B). This preparation was used to elicit polyclonal antiserum in a rabbit (cfr. Section 4). The serum had a titer of 1:128 in a double diffusion test (not shown) and was used at a 1:1000 dilution for Western blotting. The antibody produced did not react to the extract of healthy plants nor to that of plants infected with SpLV or AV-2 but did exhibit a clear specific reaction to extracts from an HdVBV-diseased plant and to the recombinant protein (Figure 6C).

## 3. Discussion

Here, we reported the characterization of a new quasi-spherical virus—including its complete nucleotide sequence—isolated from an *H. macrophylla* plant, tentatively named hydrangea vein banding virus (HdVBV).

The results obtained in this study clearly indicate that HdVBV is a new ilarvirus, showing the highest similarity to members belonging to the ilarvirus subgroup 2. Based on the indication of the International Committee on Taxonomy of Viruses [5], differences in host range, serology and genomic sequence are commonly used for species demarcation within the genus *Ilarvirus*; however, no thresholds have been defined. Although the host range study involved a limited number of inoculated hosts, some differences were observed in the host range of HdVBV compared to that reported for other ilarviruses from subgroup 2 (Figure 2) [13].

In this work, we determined the entire genomic structure of HdVBV. The region encoding the p1 protein was the most conserved when HdVBV was compared with other ilarviruses belonging to subgroup 2 (Table 1). This feature is also commonly found within members of other genera because the selection for essential functions, such as RNA replication, might be the cause of its conservation [14].

In the p3a sequence of HdVBV, some conserved motifs were identified when the sequence was compared with the same genomic region of other ilarviruses belonging to subgroup 2. Nevertheless, this genomic region showed the lowest shared identity in both nucleotide and amino acid sequences between HdVBV and the members of subgroup 2, with the exception of a high percentage of identity/similarity (i.e., 91/95%) found between HbVBV and CVV (Table 1). The variability observed for p3a could be related to the geographical or evolutionary confinement of viruses or to its involvement in specific virus–host interactions [15].

According to the International Committee on Taxonomy of Viruses [5], the species demarcation criteria in the genus ilarvirus have not yet been defined in terms of sequence similarity. However, phylogenetic analysis based on p1, p2a, p3a and p3b amino acid sequences clearly placed HdVBV as a distinct member in subgroup 2 of the genus *Ilarvirus*, along with asparagus virus 2 (AV-2), citrus leaf rugose virus (CLRV), citrus variegation virus (CVV), elm mottle virus (EMV), lilac ring mottle virus (LRMV), spinach latent virus (SpLV), Tulare apple mosaic virus (TAMV) and tomato necrotic spot virus (ToNSV) [5]. Therefore, based on the highly resolved phylogenetic tree obtained, we concluded that HdVBV is a novel virus that is distinct from the other viruses in subgroup 2.

Interestingly, a BLAST analysis of HdVBV RNA3 identified a sequence (GenBank, Acc. no. MN412730) in the database showing 97% identity with the query sequence. This sequence is annotated as “Surrounding non-legume associated ilarvirus isolate Kreis” and was identified in Germany in 2016 from an unknown weed. This correspondence further confirms: (i) the new identity of the virus studied here, (ii) that the two sequences represent two isolates of the same viral species, and (iii) that this virus is also present in Germany and perhaps in other countries as well.

As shown in Figure 1, HdVBV is able to induce ring spot symptoms in older leaves. These symptoms are very similar to those induced by distinct viruses that infect hydrangea, such as hydrangea ring spot virus (HRSV, genus *Potexvirus*, family *Alphaflexiviridae*), tomato ringspot virus (ToRSV, genus *Nepovirus*, family *Secoviridae*), tobacco ring spot virus (TRSV, genus *Nepovirus*, family *Secoviridae*), cherry leaf roll virus (CLRV, genus *Nepovirus*, family *Secoviridae*), tomato spotted wilt virus (TSWV, family *Tospoviridae*) and hydrangea chlorotic mottle virus (HdCMV, genus *Carlavirus*, family *Betaflexiviridae*). Moreover, some bacterial and fungal infections can mimic the symptoms of ring spot virus on hydrangea [16,17]. To avoid misdiagnosis based only on the presence of ring spot symptoms, and thus to investigate the real cause of hydrangea ring spot disease, it is necessary to use highly specific diagnostic systems for each of the putative ring spot disease-inducing microorganisms. In the case of HdVBV, the identity of the virus was confirmed by RT-PCR with primers derived from assembled genome sequences and by Western blot analysis with antibodies produced against a recombinant viral CP; however, we ignored the possibility of cross-reactivity with the coat protein of CVV, which had the highest percentage of shared amino acid identity in the CPs among subgroup 2 ilarviruses. These methods will be of practical use for the diagnosis, identification and control of HdVBV in hydrangea and for epidemiological studies.

## 4. Materials and Methods

### 4.1. Virus Source, Experimental Host Range and Graft Transmission

*Hydrangea macrophylla* plants of an unknown variety showing the symptoms described above were identified in a private garden in the municipality of Napoli in the Campania region of southern Italy. One symptomatic plant, referred to as HM1, was removed along with its root system and established in a pot under greenhouse conditions at the Institute for Sustainable Plant Protection of the National Research Council (IPSP-CNR), secondary section of Portici (Italy), where symptoms were recorded over the years.

Virus transmission was performed by grinding 1 g of young tobacco leaves in 4 mL of 0.03 M Na_2_HPO_4_ buffer containing 0.2% sodium diethyldithiocarbamate. Before inoculation, 75 mg mL^−1^ of carborundum and activated charcoal were added to the sap extract [18]. The slurry was gently rubbed on the first three to four true leaves of the seedlings of different herbaceous species, including *Nicotiana tabacum* ‘Xanthi nc’, *N. glutinosa, N. rustica*, *N. benthamiana*, *Chenopodium amaranticolor* and *Phaseolus vulgaris*. Five plants of each species/cultivar were inoculated and kept in an insect-proof greenhouse under natural light with the temperature controlled at 20–22 °C; the plants were checked daily for the appearance of symptoms during a period of one month. Asymptomatic inoculated plants were back-inoculated to *C. amaranticolor* to ascertain the presence or absence of latent infections.

Three buds from the symptomatic hydrangea plant were grafted onto three independent healthy hydrangea plantlets to confirm the infectivity in the original host.

### 4.2. Virus Purification and Electron Microscopy Observation

Symptomatic leaves harvested from the HM1 plant were further used to obtain a partial purified preparation of the virus as previously described in [19], with minor modifications. The final pellets were suspended in 50 mM NaCl and stored at −20 °C in the presence of 30% glycerol and trace amounts of sodium azide.

Partially purified virus preparations and plant sap from symptomatic leaves were used for dip assays (dip method). A small piece of symptomatic tissue was placed on a glass slide and a few drops of sample buffer, composed of 0.1 M Na_2_H/KH_2_PO_4_ buffer (pH 7.0), 2% (*w/v*) polyvinyl pyrrolidone (PVP, MW 11.000) and 0.2% (*w/v*) Na_2_SO_3_, were added. The leaves were gently cut into small pieces with a razor blade in the buffer and the plant sap was used to make adsorbed preparations. Carbon-coated grids of 400 mesh were incubated for 5 min with a drop of the homogenates prepared from virus-infected plant samples or from partially purified virus preparations and subsequently washed with a gentle stream of approximately 20 drops of deionized water (Milli Q) to remove buffer salts. For negative staining, 3–5 drops of a 1% uranyl acetate solution in sterile deionized water were applied. The grids were then dried by gentle tapping on a piece of Whatman paper and examined with electron microscope [20,21].

### 4.3. Determination of Coat Protein Molecular Weight, Antibody Production and Serological Assays

Two oligonucleotide primers were designed according to the nucleotide sequence of the putative coat protein (CP; accession no. OK666837). The forward (5′-*CATATG*CAACATGTCTGGTAATG-3′) and 3′ reverse (5′-*CTCGAG*GTCAATCCTCAACAACCAAG-3′) primers were synthesized and used to amplify the CP gene of the RNA extracted from the partially purified virus and infected plants via reverse transcription-polymerase chain reaction (RT-PCR). These primers resulted in the amplification of the complete CP open reading frame (ORF) and the incorporation of *Nde*I and *Xho*I restriction sites into the amplicon upstream and downstream from the CP gene, respectively. The purified PCR product was cloned into the plasmid pGEMT (Promega, Madison, WI, USA) and sequenced. The target fragment was released from the recombinant plasmid by *Nde*I and *Xho*I digestion and inserted into the corresponding sites of the pET-30a(+) plasmid (Novagen, Gibbstown, NJ, USA). This procedure resulted in a His-tag incorporated at the carboxy terminus of the CP.

The expression and purification of the proteins was performed according to the method described in [22]. Briefly, *Escherichia coli* BL21 (DE3) pLysS cells transformed with pET-30a-CP were inoculated into 3 mL of LB medium containing 100 µg/mL kanamycin and cultured at 37 °C until the OD600 reached 0.25–0.3, after which shaking was continued for an additional 30 min at 28 °C. Protein expression was then induced with 0.4 mM isopropyl-ß-d-thiogalactopyranoside (IPTG); the cell suspension was maintained for 12 h at 20 °C to increase the proportion of soluble proteins that could be recovered for affinity chromatography. The induced bacterial cells were harvested, and the pellet was resuspended in 25 mL of LB (lysis buffer; 50 mM Tris-HCl, pH 8.0, 100 mM NaCl, 5% glycerin (*v*/*v*), 1 mM imidazole and 5 mM 2-mercaptoethanol), and 1 mL of 10 mM lysozyme, 50 µL of 20 mM PMSF and 20 µg of leupeptin were subsequently added to the bacterial suspension. Next, the mixture was shaken at 50× *g* for 20 min; then, 250 µL of 8% sodium deoxycholate (NaDC) was added to the suspension before shaking on ice for another 20 min. The bacterial suspension was then sonicated, and the mixture was clarified by centrifugation at 15,500× *g* for 30 min at 4 °C. The supernatant was then loaded on a 3 mL Ni-NTA affinity column (Amersham Bioscience, Buckinghamshire, UK) and washed three times with a total of 10 mL LB. Residual proteins were then removed by washing with 50 mL of WB (wash buffer; 50 mM Tris-HCl, pH 8.0, 100 mM NaCl, 5% glycerin (*v*/*v*), 10 mM imidazole, 5 mM 2-mercaptoethanol) and EB1 (elution buffer 1; 50 mM Tris-HCl, pH 8.0, 100 mM NaCl, 5% glycerin (*v*/*v*), 50 mM imidazole and 5 mM 2-mercaptoethanol), and the washing fractions were discarded. The His-tagged CP was then purified using EB2 (elution buffer 2; 50 mM Tris-HCl, pH 8.0, 100 mM NaCl, 5% glycerin (*v*/*v*), 200 mM imidazole, 5 mM 2-mercaptoethanol). The eluted protein was concentrated by centrifugation onto an Amicon^®^ Ultra-PL30 filter unit, following the manufacturer’s instructions, and dialyzed against 0.85% NaCl overnight. The protein concentration was then determined by protein standard comparison in 12.5% SDS-polyacrylamide gel and stored at −80 °C.

One milligram of purified CP His-tagged protein was emulsified with Freund’s incomplete adjuvant for the immunization of a rabbit by intradermal and intramuscular injections at Bio-Fab research (Rome, Italy). One week after the first injection, the rabbit was further immunized with seven ear vein injections of His-tagged CP (100 µg per injection) at daily intervals. The blood was collected from the immunized rabbit 2 weeks after the final injection and the antiserum was decanted after clotting of the red blood cells.

To assess the presence of the new ilarvirus by Western blotting (WB), symptomatic leaf samples were homogenized in liquid nitrogen and the proteins were extracted in 2 × SDS protein buffer (100 mM Tris–HCl, 20% glycerol, 4% SDS, 200 mM ß-mercaptoethanol, 0.2% bromophenol blue, pH 6.8) at a ratio of 1:2 (*w*/*v*). The samples were then separated using 12.5% SDS-PAGE [23]. The fractionated protein samples were electro-transferred onto Hybond^TM^-C nitrocellulose membranes (Amersham Biosciences, Buckinghamshire, UK) using a Mini Trans-Blot (Bio-Rad, Hercules, CA, USA) at 200 mA for 1 h at 4 °C. The membranes were blocked overnight at 4 °C in PBS-Tween 20 (PBS-T) containing 5% skimmed milk powder (blocking solution). The crude polyclonal CP antiserum was then added to the blocking solution (1:1000 dilution, *v*/*v*) and the incubation was continued for 1 h at 37 °C. After three washes in PBS-T, the membranes were further treated for 1 h with protein A-alkaline phosphatase conjugate (1:3000 dilution in blocking solution, Sigma, Louis, MO, USA) at 37 °C. After another round of three washes with PBS-T, the membranes were incubated for 20 min with freshly prepared substrate solution (0.33 mg/mL NBT and 0.165 mg/mL BCIP in 0.1 M Tris–HCl buffer, pH 9.5, containing 0.1 M NaCl and 5 mM MgCl_2_).

The antiserum produced against HdVBV was tested in WB against a tomato isolate of spinach latent virus (SpLV, genus *Ilarvirus*, subgroup 2) and asparagus virus 2 (AV-2, genus *Ilarvirus*, subgroup 2), while the healthy and infected hydrangea plants were also tested in WB with a polyclonal antiserum raised against the same tomato isolate of SpLV, kindly provided by Dr. Piero Roggero (Istituto di Fitovirologia del CNR, Torino, Italy) [24]. The results were recorded using a digital camera.

### 4.4. Total RNA Extraction, Sequencing and Genome Assembly

Total RNA was extracted with the E.Z.N.A.^®^ Plant RNA Kit (Omega Bio-tek, Norcross, GA, USA) using symptomatic *H. macrophylla* and *C. quinoa* leaves, following the manufacturer’s instructions. RNA extracted from the leaves of commercial and certified healthy *H. macrophylla* plants was used as negative control in all molecular assays. The presence of viruses in the samples was ascertained via reverse transcription (RT)-PCR using different sets of specific or degenerate primers for the detection of the following virus taxa: ilarviruses [25], tospoviruses [26], carlaviruses [27], como/nepoviruses [28], closteroviruses [29], flexiviruses [30], tymo/marafiviruses [31], potyvirus [32], cucumber mosaic virus [33] and alfalfa mosaic virus [34]. PCR products of the expected sizes were obtained only with the ilarvirus PCR assays, which targeted RNA2 [25]. A BLASTn search showed no more than 85% (RNA2) sequence identity with other members of the genus Ilarvirus, with the members of subgroup 2 being the closest match. Using the available sequences of ilarviruses in subgroup 2, walking primers were designed on multialignments from the conserved regions of RNAs 1, 2 and 3 and used to further amplify the sequence of the hydrangea ilarvirus (Table S1). RT-PCR was conducted according to the manufacturer’s protocol (ImProm-II^TM^ Reverse Transcription System, Promega, Madison, WI, USA) with the following conditions: 1 cycle of 42 °C for 90 min followed by 35 cycles of 94 °C for 45 s, 52 °C for 1 min and 72 °C for 2 min, and, finally, 1 cycle of 72 °C for 10 min. Amplicons of the expected sizes were obtained using RT-PCR with most of the primer sets and were directly sequenced at Microsynth Seqlab GmbH (Göttingen, Germany) in both directions.

Based on the nucleotide sequences that were obtained, specific primers (Table S1) were redesigned to amplify fragments of the undetermined 5′-upstream and 3′-downstream regions corresponding to each segmented genome by 5′ and 3′-RACE (rapid amplification of cDNA ends), performed using the manufacturer’s protocol (2nd generation 5′/3′ RACE kit; Roche, Basel, Switzerland). For 3′-RACE-PCR, poly A tails were added to the 3′ ends of the viral RNA molecules using an A-PlusTM Poly (A) Polymerase Tailing Kit (Cellscript, Madison, WI, USA). The amplified products were purified, cloned and then sequenced as described above. All sequences obtained were first analyzed using MacVector v17.0.2 (MacVector, Inc., Apex, NC, USA) and then assembled using the Geneious 5.5 software (Biomatters Ltd., Auckland, New Zealand). In order to obtain a consensus sequence for HdVBV, multiple overlapping clones were sequenced for each genomic region encompassed by the different primer pairs to accomplish five-times coverage of each nucleotide.

### 4.5. Phylogenetic Analysis

To determine the relationship between HdVBV and other ilarviruses, phylogenetic trees were constructed for each of the viral protein ORFs using representative sequences of all member species recognized by the International Committee on Taxonomy of Viruses [5]. Multiple alignments were performed with Muscle [35], implemented in MEGA X software using the default parameters [36]. Conserved domains in the predicted viral proteins were identified with BLASTn [37] and Pfam (http://pfam.xfam.org/, accessed on 12 January 2022) [38]. The homologous sequences of cucumber mosaic virus (CMV) were included and used as outgroups for phylogenetic reconstruction. The analysis was conducted with MEGA X [36], adopting the best nucleotide substitution model, calculated specifically for each viral gene, and using the maximum likelihood method with 1000 bootstrap replicates.

## Figures and Tables

**Table 1:** Nucleotide and amino acid sequence identities of hydrangea vein banding virus and its orthologs in subgroup II of the genus *Ilarvirus* ^a^.

	CVV	EMoV	AV-2	SpLV	TAMV	ToNSV	CLRV	LRMV
Nucleotides								
RNA 1	85	86	85	79	69	70	70	67 ^b^
RNA 2	83	80	88	77	75	74	75	69
RNA 3	81	79	85	71	65	67	68	66
Amino acids								
p1	91/95 ^c^	91/95	90/95	87/92	69/80	71/84	72/84	na
p2a	87/91	82/88	90/93	74/81	70/78	63/72	67/74	60/72
p2b	79/85	68/76	85/90	67/77	72/83	69/84	66/75	54/70
p3a	91/95	35/51	34/51	33/48	30/48	35/51	75/83	48/63
p3b	76/86	75/86	81/88	66/79	58/71	63/75	60/74	37/49

^a^ Pairwise identities were calculated with ClustalW. Acronims: citrus variegation virus (CVV), elm mottle virus (EMoV), asparagus virus 2 (AV-2), spinach latent virus (SpLV), tulare apple mosaic virus (TaMV), tomato necrotic streak virus (ToNSV), citrus leaf rugose virus (CiLRV), *Lilac ring mottle virus* (LiRMV). ^b^ referred to a partial sequence available for LRMV. ^c^ identity/similarity. na = not available.

## Data Availability

Data are contained within the article or Supplementary Materials.

## Supplementary Materials

The following supporting information can be downloaded at: https://www.mdpi.com/article/10.3390/plants11070944/s1. Table S1: List of primers used in the present study.
