# Supplementary material for: A New Ilarvirus Found in French Hydrangea
Source: Plants (Basel). 2022 Mar 30;11(7):944. doi: 10.3390/plants11070944 (PMC9002526; doi:10.3390/plants11070944)
Supplement: Supplementary file 1 [file plants-11-00944-s001.zip › plants-1656361-supplementary.pdf]

Table S1. Primers used to complete the genome sequencing or for the detection of hydrangea vein banding virus (HdVBV) from symptomatic *Hydrangea macrophylla*.

	Primer	sequence 5′-3′	Reference
**RNA1**			
	Ilar2F5	TCRAYRTTYGAYAARTCNCA	
	Ilar2R9	GGTTGRTTRTGHGGRAAYTT	Untiveros et al. 2010
	HVR1Af	GTATTGTGCAGTATTATTTTAC	
	HVR1Br	CTCCWATATCRATGATBGTGGT	This study
	HVR1Bf	ACCACVATCATYGATATWGGAG	
	HVR1Cr	GACTTGATGGTATTYTTCATGG	This study
	HVR1Cf	CCATGAARGGTACCATCAAGTC	
	HVR1Dr	GARGATGGTGTGGCWGGATGTG	This study
	HVR1Df	CACATCCWGCCACACCATCYTC	
	HVR1Er	GGGCATATGGACTTRGCTTAGG	This study
	HVR1Ef	CCTAAGCYAAGTCCATATGCCC	
	HVR1Fr	GCATCTCCTTBGGAGGCATCTAA	This study
**RNA2**			
	HVR2Bf	GGTCTAGTCRGTTTGAATGYGA	
	HVR2Br	TCRCATTCAAACYGACTAGACC	This study
	HVR2Cf	CATAAGAAGGGTATMGTGATGCA	
	HVR2Cr	TGCATCACKATACCCTTCTTATG	This study
	HVR2Df	TTCGAGCTGMAGGAGTTCACCA	
	HVR2Dr	TGGTGAACTCCTKCAGCTCGAA	
	HVR2Er	GCATCTCCTTYGGAGGCATC	This study
	HV2R	GCTTTCCCGTACAAGTCGAGAG	
	HV7F	AAGTCCATATGCCCACCTTCGCT	This study
**RNA 3**			
	HV1F	CTTTCGAGACTAATAGAATTC	
	HV4R	CAAATWAGGGTATCCATCCCAC	This study
	HV2F	CTCTCGACTTGTACGGGAAAGC	
	HV6R	TTCAATAGCATTRCCAGACAT	This study
	HV3F	GTGGGATGGATACCCTWATTTG	
	HV7R	GCRAAGRTGGGCATATGGACTT	This study
	HV5F	ATGTCTGGYAATGCTATTGAA	
	HV8R	CTCTCTCCMTGAGAGAGAG	
	HV9R	GCATCTCCTTYGGAGGCACT	This study
	CP1	*CATATG*CAACATGTCTGGTAATG	
	CP2	*CTCGAG*GTCAATCCTCAACAACCAAG	This study
